# Mortality of severe pneumonia treated with methylprednisolone versus hydrocortisone: a propensity-matched analysis

**DOI:** 10.1186/s40560-025-00810-1

**Published:** 2025-07-15

**Authors:** Takuya Sato, Yusuke Sasabuchi, Ryota Inokuchi, Shotaro Aso, Hideo Yasunaga, Kent Doi

**Affiliations:** 1 Department of Emergency and Critical Care Medicine, The University of Tokyo Hospital, 7-3-1, Hongo, Bunkyo-Ku, Tokyo 1138655 Japan; 2 Department of Clinical Epidemiology and Health Economics, School of Public Health, The University of Tokyo, 7-3-1, Hongo, Bunkyo-Ku, Tokyo 1138655 Japan

**Keywords:** Severe pneumonia, Corticosteroid, Propensity score matching, In-hospital mortality, Septic shock

## Abstract

**Background:**

Corticosteroids improve the outcomes of severe pneumonia; however, the most effective type remains unknown. In this study, we compared the mortality rates of patients with severe pneumonia who were treated with methylprednisolone versus those treated with hydrocortisone.

**Methods:**

In this retrospective observational study, we utilized a nationwide Japanese Diagnosis Procedure Combination inpatient database to include adult patients with severe pneumonia who were admitted to hospitals between April 2017 and March 2022 and received either methylprednisolone or hydrocortisone. Propensity score matching was used to adjust for measured confounders, with in-hospital mortality as the primary outcome.

**Results:**

Among the 5,084 eligible patients, 623 matched pairs were analyzed. In-hospital mortality rates were 23.9% in the hydrocortisone group and 19.4% in the methylprednisolone group (risk difference [RD], 4.5%; 95% confidence interval [CI] −0.082 to 9.1; p = 0.054). Subgroup analysis of patients with shock demonstrated significantly higher mortality in the hydrocortisone group than in the methylprednisolone group (44.7% versus 30.1%; RD, 14.6%; 95% CI 1.4–27.8; p = 0.031).

**Conclusion:**

No significant difference in in-hospital mortality was observed between patients with severe pneumonia treated with methylprednisolone and those treated with hydrocortisone. Nevertheless, patients experiencing severe pneumonia-induced septic shock may derive benefits from methylprednisolone treatment.

**Supplementary Information:**

The online version contains supplementary material available at 10.1186/s40560-025-00810-1.

## Background

Community-acquired pneumonia (CAP) is the leading cause of infectious disease-related mortality globally, resulting in approximately 2 million deaths each year. In the United States, over 1.5 million adults are hospitalized annually due to CAP [1–3]. In Japan, nearly 24,000 individuals are hospitalized, with approximately 400 pneumonia-related deaths occurring daily [4, 5].

Pneumonia triggers a widespread inflammatory response throughout the lungs and the entire body. This inflammation results in impaired gas exchange, multiorgan failure, and an increased risk of mortality. Patients experiencing septic shock frequently suffer from adrenal insufficiency, which leads to refractory hypotension. Glucocorticoids, due to their potent anti-inflammatory and immunomodulatory effects, may help mitigate pneumonia-related inflammatory responses in the management of adrenal insufficiency.

Several randomized controlled trials and meta-analyses have evaluated the efficacy of corticosteroids in treating pneumonia [6–11]. Numerous studies have reported favorable outcomes and improved survival rates in patients receiving corticosteroid therapy [11]. The Society of Critical Care Medicine (SCCM) in the United States has issued guidelines recommending the administration of corticosteroids in patients with severe pneumonia [12]. In these studies and guidelines, various corticosteroids, including methylprednisolone (mPSL) and hydrocortisone, which exhibit differing glucocorticoid and mineralocorticoid effects, have been compared to placebo [13]. Methylprednisolone is commonly utilized to treat interstitial pneumonia or acute respiratory distress syndrome due to its potent glucocorticoid and anti-inflammatory properties. In contrast, hydrocortisone possesses a significant mineralocorticoid effect and is potentially indicated for use in patients with septic shock.

In clinical practice, the corticosteroids most commonly used for treating bacterial pneumonia are methylprednisolone (mPSL) and hydrocortisone. Hydrocortisone demonstrated a significant improvement in mortality over placebo in a randomized controlled trial [11], while mPSL had also been compared with placebo in some studies, and was reported to significantly reduce treatment failure in another pertinent study [7]. The SCCM guidelines also recommend these agents for CAP; however, the superiority of one drug over the other has not been established [14]. Recent evidence, including a randomized controlled trial demonstrating that hydrocortisone significantly reduced mortality compared to placebo [11], as well as a network meta-analysis indicating that hydrocortisone is more likely to result in shock reversal [15], has led us to hypothesize that hydrocortisone may offer greater benefits than mPSL. Limited evidence exists directly comparing the effectiveness of mPSL and hydrocortisone in treating severe pneumonia, particularly of bacterial origin, and whether treatment outcomes vary based on patient characteristics, such as disease severity. Therefore, this study aimed to compare in-hospital mortality due to severe pneumonia between patients receiving mPSL and those receiving hydrocortisone.

## Methods

### Data source

In this retrospective cohort study, we utilized the Japanese Diagnosis Procedure Combination (DPC) database, a nationwide administrative inpatient database previously reported [16, 17], to compare in-hospital mortality due to severe pneumonia between patients treated with mPSL and those treated with hydrocortisone. The database encompasses data from over 1,000 hospitals, representing approximately 50% of all discharges from acute care hospitals in Japan. Collected data included patient demographics (sex, age), hospitalization and discharge dates, level of consciousness upon admission, smoking index, activities of daily living (ADL), intensive care unit (ICU) admission during hospitalization, primary diagnoses, preexisting comorbidities upon admission, post-admission complications as recorded by the attending physicians according to the International Classification of Diseases, 10th revision (ICD-10) codes, with accompanying text in Japanese, procedures and their respective dates, dates of drug administration during hospitalization, and discharge status.

The database also included the A-DROP score [18], a validated measure of pneumonia severity. In a previous study, the validity of the recorded diagnoses and procedures in the database was examined, demonstrating a diagnostic specificity exceeding 96%, with sensitivity ranging from 50 to 80%. Additionally, both the specificity and sensitivity of the recorded procedures exceeded 90% [19].

This study received approval from the Institutional Review Board of the University of Tokyo (approval number: 3501-5; May 19, 2021) for clinical epidemiological research utilizing DPC data. The study was conducted in accordance with the ethical standards established by the Committee on Human Experimentation and the 1975 Declaration of Helsinki. Due to the anonymous nature of the data, the requirement for informed consent was waived. To maintain patient confidentiality, the raw data cannot be publicly disclosed. However, the data are accessible to qualified researchers upon reasonable request to the corresponding author, provided that the necessary ethical approvals have been obtained.

### Study population

Data collected from April 1, 2017, and March 31, 2022, were used. The inclusion criteria were age > 18 years, diagnosis of pneumonia of bacterial origin (ICD-10 codes J13-18), and treatment with antibiotics and corticosteroids (mPSL or hydrocortisone) within 2 days post-admission. Only patients with severe pneumonia were included, defined as fulfilling more than three of the following five A-DROP criteria [18]: (1) age ≥ 70 years for males or ≥ 75 for females; (2) dehydration (blood urea nitrogen level ≥ 21 mg/dL or physical signs of dehydration); (3) respiratory failure (SpO_2_ ≤ 90% without supplemental oxygen); (4) orientation disturbance (confusion); and (5) low blood pressure (systolic blood pressure ≤ 90 mmHg). The exclusion criteria included pregnancy, hematological malignancy, solid tumor, rheumatic disease, human immunodeficiency virus infection, major surgery under general anesthesia within 2 days post-admission, and discharge within 2 days post-admission. To focus on CAP, patients hospitalized in the same hospital within the preceding 90 days or who were transferred from another hospital were excluded. Patients who received both mPSL and hydrocortisone, as well as those without corticosteroid dosage records, were also excluded. The patients were divided into two groups based on whether they received mPSL or hydrocortisone within 2 days of admission. Only the intravenous formulations of hydrocortisone and mPSL were included, as intensive care clinicians typically consider intubation for patients with severe pneumonia and tend to avoid oral or enteral drug administration in such cases.

### Variables

Patient characteristics evaluated upon admission included sex, age, calendar year, type of hospital (tertiary or non-tertiary), smoking status (non-smoker or current/past smoker), pre-admission location (home or nursing home), presence of dementia, ADL as measured by the Barthel Index, care-needs category, ambulance use, level of consciousness, presence of asthma or chronic obstructive pulmonary disease, coronavirus disease 2019 (COVID-19), A-DROP score, Charlson Comorbidity Index (CCI), and Child–Pugh score. The CCI was calculated as previously described [20]. Additionally, we examined data on procedures and treatments administered within 2 days of admission, including supplemental oxygen therapy, high-flow nasal cannula oxygen therapy, noninvasive positive pressure ventilation, invasive mechanical ventilation, types of antibiotics used, use of multiple antibiotics, vasopressors (norepinephrine, dopamine, and dobutamine), transfusion (red blood cells, fresh frozen plasma, and platelets), dialysis, continuous hemodialysis filtration, hydrocortisone equivalent potency of administered corticosteroid agents, admission to the ICU or high-dependency care unit, extracorporeal membrane oxygenation, peripheral arterial catheter monitoring, central venous catheter insertion, and sivelestat sodium administration. These covariates were selected based on a previous study that utilized this database to investigate the treatment efficacy of corticosteroids for pneumonia [21].

The Barthel Index evaluates the performance of ADLs across 10 fundamental aspects of daily self-care and mobility, including feeding, transfer, grooming, toileting, bathing, mobility, stair climbing, dressing, bowel management, and bladder management. The total possible score is 100, with lower scores indicating greater levels of dependency [22–24]. The care needs of each dependent individual were assessed using the nationally standardized care needs certification system in Japan. Individuals were categorized into one of seven care-needs levels, each corresponding to an estimated range of daily total care minutes: support level 1 (25–31 min), support level 2 (32–49 min), care-needs level 1 (32–49 min), care-needs level 2 (50–69 min), care-needs level 3 (70–89 min), care-needs level 4 (90–109 min), and care-needs level 5 (≥ 110 min) [25, 26]. The Japan Coma Scale, widely utilized in Japan and correlating with the Glasgow Coma Scale, was employed to assess levels of consciousness [27–29]. Patients with COVID-19 was defined as individuals recorded with the ICD-10 code U07.1 (COVID-19) as a comorbidity on the day of admission. The hydrocortisone equivalent potency of mPSL was established at 5, as reported in a previous study [30].

### Outcomes

The primary outcome measure was all-cause mortality. Secondary outcomes included 28-day mortality, ventilator-free survival within 28-day of admission, vasopressor-free survival within 28-day of admission, readmission within 90-day post-discharge, length of hospital stay, and total hospitalization costs.

### Statistical analysis

Dichotomous and categorical variables were expressed as frequencies and percentages, while continuous variables were reported as medians with interquartile ranges. Missing values in categorical variables were categorized as'missing'; however, no missing values were present in continuous variables according to the eligibility criteria outlined above. To examine the differences in mortality between the two groups, one-to-one propensity score matching [31] was conducted using a logistic regression model to determine the likelihood of receiving hydrocortisone. The independent variables included all previously mentioned covariates. One-to-one nearest-neighbor matching was performed based on the estimated propensity scores, utilizing a caliper width of 0.2 standard deviations of the propensity score for the control group. A standardized mean difference (SMD) was used to assess covariate balance, with an SMD of less than 0.1 considered indicative of acceptable balance between the two groups.

Subgroup analyses were conducted for patients with and without shock. Patients classified as having shock were those who received vasopressors within 2 days of admission. These subgroup analyses utilized the propensity scores calculated in the main analysis [32].

An overlap weighting analysis was performed for the primary outcome as a sensitivity analysis. A two-tailed significance level of 0.05 was applied in all statistical analyses, which were executed using STATA/SE software (version 18; STATA Corp., College Station, TX, USA).

## Results

**Fig. 1:**
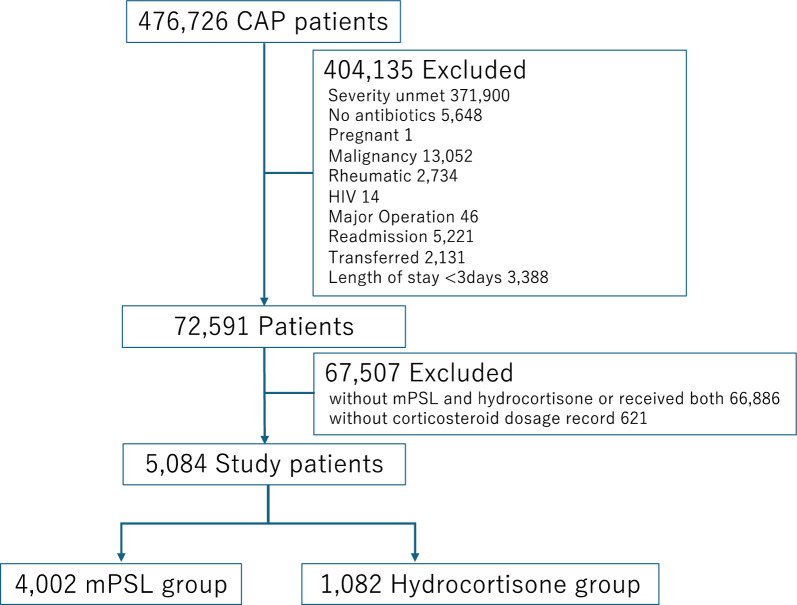
Patient selection flowchart. *CAP* community-acquired pneumonia, *HIV* human immunodeficiency virus, *mPSL* methylprednisolone

We identified 476,726 patients hospitalized for pneumonia between April 1, 2017, and March 31, 2022, from the database. Among these, 471,642 patients were excluded, resulting in a study population of 5,084 patients with severe pneumonia (including 4,002 patients in the mPSL group and 1,082 patients in the hydrocortisone group). Notably, only four patients with COVID-19 were included in the study. The patient selection flowchart is presented in Fig. 1. The mean age of the patients was 80.6 years, with 74.2% being male. The baseline patient characteristics and comorbidities are detailed in Table 1 and Supplemental Table 1. The unadjusted in-hospital mortality rates were 22.3% in the mPSL group and 28.9% in the hydrocortisone group.

**Table 1:** Patients’ baseline characteristics and comorbidities

	Before matching				After matching		
	Total	mPSL	Hydrocortisone	Standardized difference	mPSL	Hydrocortisone	Standardized difference
	N = 5,084	N = 4,002	N = 1,082		N = 623	N = 623	
Age	80.6 (9.0)	81.1 (8.4)	78.8 (10.8)	−0.23	80.6	81.4	0.094
Male	3,772 (74.2%)	3,003 (75.0%)	769 (71.1%)	−0.089	71%	70%	−0.021
Fiscal year							
2017	1,319 (25.9%)	1,024 (25.6%)	295 (27.3%)	0.038	29%	27%	−0.039
2018	1,308 (25.7%)	1,041 (26.0%)	267 (24.7%)	−0.031	28%	26%	−0.033
2019	1,149 (22.6%)	896 (22.4%)	253 (23.4%)	0.024	20%	25%	0.13
2020	681 (13.4%)	545 (13.6%)	136 (12.6%)	−0.031	13%	12%	−0.039
2021	627 (12.3%)	496 (12.4%)	131 (12.1%)	−0.009	11%	10%	−0.032
Hospital volume	140.5 (109.6)	142.8 (113.5)	132.1 (93.6)	−0.103	139	131	−0.075
Smoking							
Non-smoker	2,145 (42.2%)	1,613 (40.3%)	532 (49.2%)	0.18	45%	49%	0.074
Smoker	2,206 (43.4%)	1,839 (46.0%)	367 (33.9%)	−0.25	42%	35%	−0.14
Missing	733 (14.4%)	550 (13.7%)	183 (16.9%)	0.088	13%	16%	0.091
JCS							
0	3,166 (62.3%)	2,663 (66.5%)	503 (46.5%)	−0.41	57%	58%	0.029
1–3	1,294 (25.5%)	986 (24.6%)	308 (28.5%)	0.087	28%	26%	−0.051
10–30	323 (6.4%)	195 (4.9%)	128 (11.8%)	0.25	9%	9%	0.022
100–300	301 (5.9%)	158 (3.9%)	143 (13.2%)	0.34	7%	7%	0.006
A-DROP score							
3	3,566 (70.1%)	2,973 (74.3%)	593 (54.8%)	−0.42	65%	68%	0.075
4	1,206 (23.7%)	872 (21.8%)	334 (30.9%)	0.21	27%	26%	−0.044
5	312 (6.1%)	157 (3.9%)	155 (14.3%)	0.37	8%	6%	−0.063
Asthma	1,021 (20.1%)	887 (22.2%)	134 (12.4%)	−0.26	20%	19%	−0.016
COPD	648 (12.7%)	571 (14.3%)	77 (7.1%)	−0.23	11%	9%	−0.063
Vasopressor							
Noradrenaline	734 (14.4%)	287 (7.2%)	447 (41.3%)	0.87	20%	13%	−0.19
Dopamine	169 (3.3%)	107 (2.7%)	62 (5.7%)	0.15	4%	3%	−0.043
Dobutamine	132 (2.6%)	54 (1.3%)	78 (7.2%)	0.29	3%	2%	−0.077
Supplemental oxygen	3,552 (69.9%)	2,926 (73.1%)	626 (57.9%)	−0.33	67%	73%	0.12
NHF	388 (7.6%)	329 (8.2%)	59 (5.5%)	−0.11	6%	6%	−0.007
NPPV	97 (1.9%)	83 (2.1%)	14 (1.3%)	−0.061	3%	1%	−0.099
Mechanical Ventilation	1,289 (25.4%)	886 (22.1%)	403 (37.2%)	0.34	25%	20%	−0.11
Antibiotics							
Multiple antibiotics	2,214 (43.5%)	1,669 (41.7%)	545 (50.4%)	0.17	43%	39%	−0.065
Ampicillin/sulbactam	1,327 (26.1%)	1,038 (25.9%)	289 (26.7%)	0.018	32%	31%	−0.024
Tazobactam/piperacillin or Tazobactam/ceftolozane	1,250 (24.6%)	951 (23.8%)	299 (27.6%)	0.089	22%	23%	0.031
Third-generation cephalosporin, poor activity against Pseudomonas	1,672 (32.9%)	1,348 (33.7%)	324 (29.9%)	−0.080	32%	31%	−0.021
Carbapenem	986 (19.4%)	693 (17.3%)	293 (27.1%)	0.24	18%	17%	−0.021
Fluoroquinolone	742 (14.6%)	585 (14.6%)	157 (14.5%)	−0.003	13%	12%	−0.024
Macrolide	981 (19.3%)	762 (19.0%)	219 (20.2%)	0.030	18%	17%	−0.021
Anti-MRSA drug	177 (3.5%)	69 (1.7%)	108 (10.0%)	0.36	4%	4%	−0.008
Hydrocortisone equivalent corticosteroid volume	1046.4 (1500.3)	1265.9 (1621.3)	234.6 (133.8)	−0.90	276	252	−0.16
ICU	676 (13.3%)	359 (9.0%)	317 (29.3%)	0.54	15%	11%	−0.11
HDU	1,131 (22.2%)	875 (21.9%)	256 (23.7%)	0.043	23%	20%	−0.078

*mPSL* methylprednisolone, *JCS* Japan coma scale, *COPD* chronic obstructive pulmonary disease, *NHF* nasal high flow therapy, *NPPV* noninvasive positive pressure ventilation, *ICU* intensive care unit, *HDU* high dependency unit

Following a 1:1 propensity score matching, 623 matched pairs were identified. Most background variables were well-balanced between the mPSL and hydrocortisone groups (Table 1 and Supplementary Table 1). However, certain variables showed significant standardized differences. In the matched cohort, the difference in in-hospital mortality between the hydrocortisone and mPSL groups was not significant (23.9% versus 19.4%; risk difference [RD], 4.5%; 95% confidence interval [CI] −0.082 to 9.1; p = 0.054). Similarly, no significant difference were observed in the 28-day mortality (18.8% in the hydrocortisone group vs. 15.6% in the mPSL group; RD, 3.2%; 95% CI −0.98 to 7.4; p = 0.13), ventilator-free days (22.1 vs. 21.8 d; RD, −0.66 d; 95% CI −1.7 to 0.38; p = 0.21), vasopressor-free days (23.6 vs. 23.7 d; RD, −0.13 d; 95% CI −1.1 to 0.81; p = 0.78), length of hospital stay (26.1 vs. 23.5 d; RD, 2.5 d; 95% CI −0.29 to 5.3; p = 0.078), readmission within 90-day (2.2% vs. 2.4%; RD, −0.16%; 95% CI −1.8 to 1.5; p = 0.85), and total hospitalization costs (1,212,000 vs. 1,165,000 Japanese yen; RD, 47,000; 95% CI −73,000 to 168,000 yen; p = 0.44) (Table 2).

**Table 2:** Outcomes and their differences between methylprednisolone and hydrocortisone after propensity score matching

	mPSL N = 623	Hydrocortisone N = 623	Difference	95% CI	P
In-hospital mortality, n (%)	121 (19.4)	149 (23.9)	4.5	−0.082 to 9.1	0.054
28-day mortality, n (%)	97 (15.6)	117 (18.8)	3.2	−0.98 to 7.4	0.13
Ventilator-free days	22.8	22.1	−0.66	−1.7 to 0.38	0.21
Vasopressor-free days	23.7	23.6	−0.13	−1.1 to 0.81	0.78
Length of hospital stay, days	23.5	26.1	2.5	−0.29 to 5.3	0.078
Readmission, n (%)	15 (2.4)	14 (2.2)	−0.16	−1.8 to 1.5	0.85
Total cost, thousand JPY	1165	1212	47	−73 to 168	0.44

*mPSL* methylprednisolone, *JPY* Japanese Yen, *CI* confidential interval

In subgroup analyses, 103 and 493 matched pairs of patients with and without shock, respectively, were identified. Some variables, including the A-DROP score and the use of mechanical ventilation, showed significant imbalance in the subgroup of patients with shock. The characteristics and comorbidities are shown in Supplementary Table 2 and Supplementary Table 3. Among patients with shock, both in-hospital and 28-day mortality rates were significantly higher in the hydrocortisone group than in the mPSL group (in-hospital mortality: 44.7% vs. 30.1%; RD, 14.6%; 95% CI 1.4–27.8; p = 0.031; 28-day mortality: 37.9% vs. 22.3%; RD, 15.5%; 95% CI 3.1–28.0; p = 0.015). Additionally, ventilator-free days (12.9 vs. 17.2 d; RD, −4.3 d; 95% CI −7.3 to −1.4; p = 0.0037) and vasopressor-free days (15.2 vs. 18.4 d; RD, −3.3 d; 95% CI −6.2 to −0.31; p = 0.030) were significantly shorter in the hydrocortisone group than in the mPSL group. No significant differences in outcomes were observed among the subgroup of patients without shock. The results of the subgroup analyses are shown in Table 3.

**Table 3:** Results of subgroup analyses: patients with shock and without shock

	mPSL	Hydrocortisone	Difference	95%CI	P
Patients with shock	*N = 103*	*N = 103*			
In-hospital mortality, n (%)	31 (30.1)	46 (44.7)	14.6	1.4 to 27.8	0.031
28-day mortality, n (%)	23 (22.3)	39 (37.9)	15.5	3.1 to 28.0	0.015
Ventilator-free days	17.2	12.9	−4.3	−7.3 to −1.4	0.0037
Vasopressor-free days	18.4	15.2	−3.3	−6.2 to −0.31	0.030
Length of hospital stay, days	30.3	28.9	−1.4	−10.3 to 7.4	0.75
Readmission, n (%)	2 (1.9)	2 (1.9)	0	−3.8 to 3.8	1.00
Total cost, thousand JPY	1912	2105	193	−238 to 625	0.38
Patients without shock	*N = 493*	*N = 493*			
In-hospital mortality, n (%)	80 (16.2)	102 (20.7)	4.5	−0.38 to 9.3	0.071
28-day mortality, n (%)	72 (14.6)	78 (15.8)	1.2	−3.3 to 5.7	0.60
Ventilator-free days	24.3	23.7	−0.55	−1.6 to 0.47	0.29
Vasopressor-free days	25.4	25.0	−0.37	−1.2 to 0.50	0.40
Length of hospital stay, days	22.6	25.1	2.5	−0.42 to 5.4	0.093
Readmission, n (%)	14 (2.8)	12 (2.4)	−0.41	−2.4 to 1.6	0.690
Total cost, thousand JPY	987	1040	53	−47 to 152	0.30

*mPSL* methylprednisolone, *JPY* Japanese Yen, *CI* confidential interval

A sensitivity analysis using overlap weighting revealed significantly higher odds of in-hospital mortality in the hydrocortisone group than in the mPSL group (odds ratio, 1.29; 95% CI 1.03–1.62; p = 0.027).

## Discussion

In this nationwide observational study, we found no significant difference in in-hospital mortality between the mPSL and hydrocortisone groups in patients with severe pneumonia. These results were consistent with those observed in patients without shock. Our findings revealed no significant difference in the secondary outcomes between the groups. However, the in-hospital and 28-day mortality rates were significantly lower in the mPSL group than in the hydrocortisone group among patients with shock.

Currently, no studies have been conducted to compare short-term mortality between mPSL and hydrocortisone in patients with severe pneumonia. Previous research has primarily focused on the comparative effects of different corticosteroid types on COVID-19 pneumonia [33, 34]. A network meta-analysis [33] indicated a lower, albeit non-significant, mortality rate with mPSL than other corticosteroids. Similarly, a small, three-arm randomized trial comparing dexamethasone, mPSL, and hydrocortisone [11] found no significant differences in mortality among the groups. Our findings are consistent with those of prior studies, suggesting that despite the differing balance of glucocorticoids and mineralocorticoids between the two drug groups, their impact on clinical outcomes may be minimal [32, 33].

In contrast, a network meta-analysis aimed at comparing corticosteroid usage in patients with septic shock [15] indicated that hydrocortisone administration was more effective in achieving shock reversal than mPSL. However, this did not correspond to a statistically significant difference in overall mortality rates. Our findings differ from those of the aforementioned study. In our subgroup analysis of patients experiencing shock, we observed that vasopressor-free days were shorter, and in-hospital mortality was higher in the hydrocortisone group than in the mPSL group. This discrepancy may be attributed to the underlying etiology of the septic shock. Patients with septic shock resulting from pneumonia may derive greater benefit from the stronger anti-inflammatory effects of mPSL, which could lead to more stable circulation.

The significant decrease in the number of patients after 2020 likely reflects a reduction in cases of bacterial pneumonia, attributed to improved public hygiene practices, such as universal masking and frequent handwashing following the COVID-19 pandemic [35]. Since dexamethasone, rather than hydrocortisone or mPSL, is the corticosteroid predominantly utilized for treating COVID-19 pneumonia, the influence of the COVID-19 pandemic on the selection between hydrocortisone and mPSL is presumed to be minimal.

Our results indicated minimal safety concerns regarding corticosteroid selection for patients with severe pneumonia, with no significant differences observed in ventilator-free days, 90-day readmission rates, and length of hospital stay. While corticosteroid users may occasionally develop additional infections due to immunosuppression, leading to longer hospital stays or readmissions, the adverse effects of both medications are comparable and consistent with the findings of a previous study [34]. Based on our findings and the existing literature, clinicians treating patients with severe pneumonia may opt for either mPSL or hydrocortisone at their discretion, taking into account the patient's specific condition. However, for patients experiencing septic shock due to severe pneumonia, mPSL may be the preferable option over hydrocortisone. Further prospective studies are warranted to validate our results.

This study has some limitations. First, despite employing propensity score matching, residual imbalances persisted for variables, such as smoking status, mechanical ventilation, and noradrenaline administration. These imbalances may act as confounders, although the direction of these imbalances generally favored the hydrocortisone group, potentially leading to an underestimation of the benefits of mPSL. Subgroup analyses, particularly among patients with shock, revealed greater imbalances; therefore, the results should be interpreted with caution. Second, unmeasured confounders may have been present due to the study's retrospective observational nature. For instance, the database lacked critical data on vital signs upon admission and the dosage of vasopressors. While the A-DROP score and vasopressor use were balanced through propensity score matching, blood pressure and respiratory rate may have been well-balanced between the groups.

Additionally, data on laboratory tests and mechanical ventilation settings were unavailable, contributing to potential confounding. In the subgroup analyses for patients with shock, it was also unclear whether the shock was septic or hypovolemic. Third, the diagnosis of pneumonia was based on administrative data and was not corroborated through radiological, biological, or pathological examinations, which may introduce diagnostic uncertainty. Fourth, pneumonia severity was assessed using the A-DROP score. While this score has been validated in a limited number of studies [18, 36], the pneumonia severity index [11, 37, 38] or CURB-65 score [39, 40] is more widely used for evaluating pneumonia severity. Fifth, since this study does not examine the therapeutic effect of corticosteroids on severe bacterial pneumonia, but compared hydrocortisone with mPSL, it does not demonstrate that corticosteroids have any significant effects on severe bacterial pneumonia. Lastly, the database comprised a Japanese population, and the proportion of analyzed patients among the overall cohort with severe pneumonia was low. Consequently, the generalizability of the results to other populations remains uncertain.

## Conclusion

This study demonstrated no significant differences in short-term outcomes regarding mortality between mPSL and hydrocortisone in patients with severe pneumonia. If clinicians are considering the administration of corticosteroids for patients with severe pneumonia and shock, mPSL may be the preferable option over hydrocortisone.

## Supplementary Information

Supplementary material 1.

## Data Availability

No datasets were generated or analysed during the current study.

## Abbreviations

mPSL: Methylprednisolone
RD: Risk difference
CI: Confidence interval
CAP: Community-acquired pneumonia
SCCM: Society of Critical Care Medicine
ADL: Activities of daily living
ICU: Intensive care unit
ICD-10: International Classification of Diseases, 10th revision
DPC: Diagnosis procedure combination
COVID-19: Coronavirus disease 2019
CCI: Charlson comorbidity index
SMD: Standardized mean difference
