# Supplementary material for: Mortality of severe pneumonia treated with methylprednisolone versus hydrocortisone: a propensity-matched analysis
Source: J Intensive Care. 2025 Jul 15;13:39. doi: 10.1186/s40560-025-00810-1 (PMC12261853; doi:10.1186/s40560-025-00810-1)
Supplement: Supplementary file 1 — Supplementary material 1. [file 40560_2025_810_MOESM1_ESM.docx]

Supplementary Table 1. The patients baseline characteristics and comorbidities.

|  |  | **Before matching** | | | |  | **After matching** | | |
| --- | --- | --- | --- | --- | --- | --- | --- | --- | --- |
|  |  | **Total** | **mPSL** | **Hydrocortisone** | Standardized difference |  | **mPSL** | **Hydrocortisone** | Standardized difference |
|  |  | N=5,084 | N=4,002 | N=1,082 |  |  | N=623 | N=623 |  |
| **Tertiary Hospital** |  | 1,935 (38.1%) | 1,425 (35.6%) | 510 (47.1%) | 0.24 |  | 38% | 36% | -0.053 |
| **Transferred from** | Home | 4,863 (95.7%) | 3,844 (96.1%) | 1,019 (94.2%) | -0.087 |  | 93% | 94% | 0.040 |
|  | Nursing home | 214 (4.2%) | 152 (3.8%) | 62 (5.7%) | 0.091 |  | 6% | 6% | -0.014 |
|  | others | 7 (0.1%) | 6 (0.1%) | 1 (0.1%) | -0.017 |  | 1.0% | 0.0% | -0.11 |
| **Barthel Index** |  |  |  |  |  |  |  |  |  |
| Feeding | Independent | 1,816 (35.7%) | 1,303 (32.6%) | 513 (47.4%) | 0.31 |  | 40% | 37% | -0.079 |
|  | Partial dependent | 1,062 (20.9%) | 879 (22.0%) | 183 (16.9%) | -0.13 |  | 20% | 22% | 0.063 |
|  | Dependent | 1,841 (36.2%) | 1,563 (39.1%) | 278 (25.7%) | -0.29 |  | 32% | 34% | 0.1 |
|  | missing | 365 (7.2%) | 257 (6.4%) | 108 (10.0%) | 0.13 |  | 8% | 7% | -0.042 |
| Transfer | Independent | 2,308 (45.4%) | 1,674 (41.8%) | 634 (58.6%) | 0.34 |  | 51% | 48% | -0.064 |
|  | Minor help | 385 (7.6%) | 310 (7.7%) | 75 (6.9%) | -0.031 |  | 7% | 8% | 0.0 |
|  | Major help | 1,169 (23.0%) | 1,003 (25.1%) | 166 (15.3%) | -0.24 |  | 20% | 22% | 0.051 |
|  | Dependent | 1,093 (21.5%) | 920 (23.0%) | 173 (16.0%) | -0.18 |  | 18% | 19% | 0.025 |
|  | Missing | 129 (2.5%) | 95 (2.4%) | 34 (3.1%) | 0.047 |  | 3% | 2% | -0.010 |
| Grooming | Independent | 3,297 (64.9%) | 2,505 (62.6%) | 792 (73.2%) | 0.23 |  | 68% | 69% | 0.014 |
|  | Dependent | 1,563 (30.7%) | 1,332 (33.3%) | 231 (21.3%) | -0.27 |  | 29% | 28% | -0.021 |
|  | Missing | 224 (4.4%) | 165 (4.1%) | 59 (5.5%) | 0.062 |  | 3% | 4% | 0.00 |
| Toilet | Independent | 2,482 (48.8%) | 1,817 (45.4%) | 665 (61.5%) | 0.33 |  | 55% | 52% | -0.045 |
|  | Partial dependent | 1,182 (23.2%) | 987 (24.7%) | 195 (18.0%) | -0.16 |  | 20% | 25% | 0.12 |
|  | Dependent | 1,239 (24.4%) | 1,067 (26.7%) | 172 (15.9%) | -0.27 |  | 22% | 19% | -0.071 |
|  | missing | 181 (3.6%) | 131 (3.3%) | 50 (4.6%) | 0.069 |  | 3% | 3% | 0.00 |
| Bathing | Independent | 3,417 (67.2%) | 2,639 (65.9%) | 778 (71.9%) | 0.13 |  | 71% | 71% | 0.004 |
|  | Dependent | 1,109 (21.8%) | 948 (23.7%) | 161 (14.9%) | -0.23 |  | 18% | 18% | -0.017 |
|  | Missing | 558 (11.0%) | 415 (10.4%) | 143 (13.2%) | 0.088 |  | 11% | 11% | 0.015 |
| Mobility | Independent | 2,656 (52.2%) | 1,973 (49.3%) | 683 (63.1%) | 0.28 |  | 55% | 57% | 0.023 |
|  | Walk with help | 340 (6.7%) | 293 (7.3%) | 47 (4.3%) | -0.13 |  | 7% | 6% | -0.033 |
|  | Wheelchair | 619 (12.2%) | 518 (12.9%) | 101 (9.3%) | -0.12 |  | 10% | 13% | 0.085 |
|  | Immobile | 1,118 (22.0%) | 960 (24.0%) | 158 (14.6%) | -0.24 |  | 20% | 17% | -0.070 |
|  | Missing | 351 (6.9%) | 258 (6.4%) | 93 (8.6%) | 0.082 |  | 7% | 7% | -0.012 |
| Stairs | Independent | 2,845 (56.0%) | 2,146 (53.6%) | 699 (64.6%) | 0.23 |  | 61% | 59% | -0.043 |
|  | Partial dependent | 619 (12.2%) | 506 (12.6%) | 113 (10.4%) | -0.069 |  | 10% | 15% | 0.14 |
|  | Dependent | 1,027 (20.2%) | 886 (22.1%) | 141 (13.0%) | -0.24 |  | 18% | 15% | -0.069 |
|  | missing | 593 (11.7%) | 464 (11.6%) | 129 (11.9%) | 0.010 |  | 11% | 11% | 0.005 |
| Dressing | Independent | 2,543 (50.0%) | 1,860 (46.5%) | 683 (63.1%) | 0.34 |  | 56% | 53% | -0.058 |
|  | Partial dependent | 1,211 (23.8%) | 1,024 (25.6%) | 187 (17.3%) | -0.20 |  | 22% | 24% | 0.057 |
|  | Dependent | 1,188 (23.4%) | 1,008 (25.2%) | 180 (16.6%) | -0.21 |  | 20% | 20% | 0.008 |
|  | missing | 142 (2.8%) | 110 (2.7%) | 32 (3.0%) | 0.013 |  | 3% | 3% | 0.010 |
| Bowel | Independent | 2,243 (44.1%) | 1,627 (40.7%) | 616 (56.9%) | 0.33 |  | 49% | 47% | -0.055 |
|  | Partial dependent | 735 (14.5%) | 599 (15.0%) | 136 (12.6%) | -0.070 |  | 14% | 16% | 0.072 |
|  | Dependent | 1,878 (36.9%) | 1,607 (40.2%) | 271 (25.0%) | -0.33 |  | 33% | 33% | -0.010 |
|  | missing | 228 (4.5%) | 169 (4.2%) | 59 (5.5%) | 0.057 |  | 4% | 4% | 0.033 |
| Bladder | Independent | 2,264 (44.5%) | 1,646 (41.1%) | 618 (57.1%) | 0.32 |  | 50% | 47% | -0.061 |
|  | Partial dependent | 756 (14.9%) | 615 (15.4%) | 141 (13.0%) | -0.067 |  | 14% | 17% | 0.084 |
|  | Dependent | 1,847 (36.3%) | 1,583 (39.6%) | 264 (24.4%) | -0.33 |  | 32% | 31% | -0.010 |
|  | missing | 217 (4.3%) | 158 (3.9%) | 59 (5.5%) | 0.071 |  | 4% | 4% | 0.025 |
| **Care-Needs** | None | 827 (16.3%) | 667 (16.7%) | 160 (14.8%) | -0.052 |  | 13% | 13% | 0.005 |
|  | Support level 1 | 57 (1.1%) | 49 (1.2%) | 8 (0.7%) | -0.049 |  | 1% | 1% | -0.051 |
|  | Support level 2 | 77 (1.5%) | 62 (1.5%) | 15 (1.4%) | -0.014 |  | 1% | 2% | 0.054 |
|  | Care-needs level 1 | 87 (1.7%) | 70 (1.7%) | 17 (1.6%) | -0.014 |  | 1% | 1% | -0.0 |
|  | Care-needs level 2 | 118 (2.3%) | 91 (2.3%) | 27 (2.5%) | 0.015 |  | 3% | 3% | -0.0 |
|  | Care-needs level 3 | 61 (1.2%) | 39 (1.0%) | 22 (2.0%) | 0.087 |  | 1% | 1% | 0.079 |
|  | Care-needs level 4 | 59 (1.2%) | 41 (1.0%) | 18 (1.7%) | 0.056 |  | 1% | 1% | 0.0 |
|  | Care-needs level 5 | 32 (0.6%) | 21 (0.5%) | 11 (1.0%) | 0.056 |  | 1% | 1% | -0.017 |
|  | Missing | 3,766 (74.1%) | 2,962 (74.0%) | 804 (74.3%) | 0.007 |  | 78% | 77% | -0.023 |
| **Charlson Comorbidity Index** |  |  |  |  |  |  |  |  |  |
| Myocardial Infarction |  | 108 (2.1%) | 89 (2.2%) | 19 (1.8%) | -0.033 |  | 2% | 2% | 0.011 |
| Congestive Heart Failure |  | 1,472 (29.0%) | 1,186 (29.6%) | 286 (26.4%) | -0.071 |  | 30% | 29% | -0.011 |
| Peripheral vascular disease |  | 93 (1.8%) | 75 (1.9%) | 18 (1.7%) | -0.016 |  | 1% | 2% | 0.026 |
| Cerebrovascular disease |  | 431 (8.5%) | 323 (8.1%) | 108 (10.0%) | 0.067 |  | 12% | 10% | -0.062 |
| Dementia |  | 389 (7.7%) | 286 (7.1%) | 103 (9.5%) | 0.086 |  | 11% | 11% | 0.0 |
| Chronic pulmonary disease |  | 2,239 (44.0%) | 1,977 (49.4%) | 262 (24.2%) | -0.54 |  | 36% | 36% | -0.007 |
| Peptic ulcer disease |  | 148 (2.9%) | 125 (3.1%) | 23 (2.1%) | -0.062 |  | 3% | 2% | -0.053 |
| Mild liver disease |  | 114 (2.2%) | 75 (1.9%) | 39 (3.6%) | 0.11 |  | 3% | 3% | 0.00 |
| Diabetes without complication |  | 916 (18.0%) | 747 (18.7%) | 169 (15.6%) | -0.081 |  | 16% | 17% | 0.021 |
| Diabetes with complication |  | 149 (2.9%) | 111 (2.8%) | 38 (3.5%) | 0.042 |  | 3% | 3% | 0.018 |
| Paralysis |  | 7 (0.1%) | 3 (0.1%) | 4 (0.4%) | 0.063 |  | 0.0% | 0.0% | -0.0 |
| Renal disease |  | 340 (6.7%) | 263 (6.6%) | 77 (7.1%) | 0.022 |  | 8% | 8% | 0.0 |
| Severe liver disease |  | 8 (0.2%) | 2 (0.0%) | 6 (0.6%) | 0.092 |  | 0.0% | 0.0% | 0.057 |
| **Child-Pugh score** | >9 | 6 (0.1%) | 1 (0.0%) | 5 (0.5%) | 0.089 |  | 0.0% | 0.0% | 0.0 |
| **Dialysis** |  | 36 (0.7%) | 27 (0.7%) | 9 (0.8%) | 0.018 |  | 1.0% | 1% | -0.0 |
| **CHDF** |  | 81 (1.6%) | 22 (0.5%) | 59 (5.5%) | 0.29 |  | 1% | 0% | -0.072 |
| **Transfusion** |  |  |  |  |  |  |  |  |  |
| RBC |  | 156 (3.1%) | 79 (2.0%) | 77 (7.1%) | 0.25 |  | 2% | 3% | 0.052 |
| FFP |  | 51 (1.0%) | 13 (0.3%) | 38 (3.5%) | 0.23 |  | 0% | 0% | 0.0 |
| Platelet |  | 40 (0.8%) | 11 (0.3%) | 29 (2.7%) | 0.20 |  | 0% | 1% | 0.021 |
| **Antibiotics** |  |  |  |  |  |  |  |  |  |
| multiple antibiotics |  | 2,214 (43.5%) | 1,669 (41.7%) | 545 (50.4%) | 0.17 |  | 43% | 39% | -0.065 |
| Penicillin |  | 26 (0.5%) | 18 (0.4%) | 8 (0.7%) | 0.038 |  | 0.0% | 1.0% | 0.021 |
| Broad-spectrum penicillin |  | 40 (0.8%) | 28 (0.7%) | 12 (1.1%) | 0.043 |  | 1% | 1% | -0.061 |
| First-generation cephalosporin |  | 11 (0.2%) | 7 (0.2%) | 4 (0.4%) | 0.037 |  | 0% | 0.0% | 0.0 |
| Second-generation cephalosporin |  | 27 (0.5%) | 19 (0.5%) | 8 (0.7%) | 0.034 |  | 1% | 1% | 0.017 |
| Third-generation cephalosporin,  good activity against Pseudomonas |  | 72 (1.4%) | 62 (1.5%) | 10 (0.9%) | -0.057 |  | 1% | 2% | 0.013 |
| Fourth-generation cephalosporin |  | 109 (2.1%) | 91 (2.3%) | 18 (1.7%) | -0.044 |  | 2% | 2% | -0.012 |
| Aminoglycoside |  | 11 (0.2%) | 9 (0.2%) | 2 (0.2%) | -0.009 |  | 0.0% | 0.0% | -0.057 |
| Tetracycline |  | 162 (3.2%) | 135 (3.4%) | 27 (2.5%) | -0.052 |  | 4% | 3% | -0.009 |
| Sulfamethoxazole-Trimethoprim |  | 132 (2.6%) | 117 (2.9%) | 15 (1.4%) | -0.11 |  | 2% | 2% | 0.0 |
| Antifungal drug |  | 89 (1.8%) | 64 (1.6%) | 25 (2.3%) | 0.051 |  | 3% | 2% | -0.067 |
| **ECMO** |  | 20 (0.4%) | 2 (0.0%) | 18 (1.7%) | 0.18 |  | 0.0% | 0% | -0.057 |
| **Arterial catheter** |  | 846 (16.6%) | 424 (10.6%) | 422 (39.0%) | 0.70 |  | 20% | 14% | -0.14 |
| **Central venous catheter** |  | 685 (13.5%) | 323 (8.1%) | 362 (33.5%) | 0.66 |  | 15% | 9% | -0.20 |
| **Sivelestat** |  | 48 (0.9%) | 40 (1.0%) | 8 (0.7%) | -0.028 |  | 1% | 0% | -0.64 |

mPSL; methylprednisolone, CHDF; continuous hemodialysis filtration, RBC; red blood cell, FFP; fresh frozen plazma, ECMO; extracorporeal membrane oxygenation.

Supplementary Table 2. Baseline characteristics and comorbidities of the subgroup of patients with shock

|  |  | **Before matching** | | | |  | **After matching** | | |
| --- | --- | --- | --- | --- | --- | --- | --- | --- | --- |
|  |  | Total | mPSL | Hydrocortisone | Standardised difference |  | mPSL | Hydrocortisone | Standardised difference |
|  |  | N=847 | N=377 | N=470 |  |  | N=103 | N=103 |  |
| **Age** |  | 75.4 (11.5) | 76.6 (10.6) | 74.5 (12.0) | -0.183 |  | 75.4 | 76.3 | 0.079 |
| **male** |  | 653 (77.1%) | 288 (76.4%) | 365 (77.7%) | 0.030 |  | 75% | 75% | -0.0 |
| **Fiscal year** |  |  |  |  |  |  |  |  |  |
|  | 2017 | 202 (23.8%) | 87 (23.1%) | 115 (24.5%) | 0.033 |  | 27% | 18% | -0.208 |
|  | 2018 | 195 (23.0%) | 92 (24.4%) | 103 (21.9%) | -0.059 |  | 24% | 26% | 0.044 |
|  | 2019 | 185 (21.8%) | 78 (20.7%) | 107 (22.8%) | 0.050 |  | 19% | 29% | 0.227 |
|  | 2020 | 142 (16.8%) | 72 (19.1%) | 70 (14.9%) | -0.112 |  | 17% | 15% | -0.053 |
|  | 2021 | 123 (14.5%) | 48 (12.7%) | 75 (16.0%) | 0.092 |  | 13% | 12% | -0.030 |
| **Hospital Volume** |  | 141.9 (114.3) | 147.7 (129.3) | 137.2 (100.7) | -0.090 |  | 155 | 158 | 0.022 |
| **Smoking** | Non-Smoker | 352 (41.6%) | 160 (42.4%) | 192 (40.9%) | -0.032 |  | 35% | 41% | 0.12 |
|  | Smoker | 327 (38.6%) | 156 (41.4%) | 171 (36.4%) | -0.103 |  | 50% | 42% | -0.175 |
|  | Missing | 168 (19.8%) | 61 (16.2%) | 107 (22.8%) | 0.167 |  | 15% | 17% | 0.079 |
| **JCS** | 0 | 282 (33.3%) | 151 (40.1%) | 131 (27.9%) | -0.259 |  | 37% | 34% | -0.061 |
|  | 1-3 | 253 (29.9%) | 111 (29.4%) | 142 (30.2%) | 0.017 |  | 27% | 27% | -0.0 |
|  | 10-30 | 126 (14.9%) | 46 (12.2%) | 80 (17.0%) | 0.137 |  | 14% | 17% | 0.107 |
|  | 100-300 | 186 (22.0%) | 69 (18.3%) | 117 (24.9%) | 0.161 |  | 22% | 21% | -0.023 |
| **A-DROP score** | 3 | 318 (37.5%) | 160 (42.4%) | 158 (33.6%) | -0.182 |  | 34% | 39% | 0.101 |
|  | 4 | 332 (39.2%) | 148 (39.3%) | 184 (39.1%) | -0.002 |  | 48% | 36% | -0.237 |
|  | 5 | 197 (23.3%) | 69 (18.3%) | 128 (27.2%) | 0.214 |  | 18% | 25% | 0.164 |
| **Asthma** |  | 57 (6.7%) | 40 (10.6%) | 17 (3.6%) | -0.274 |  | 11% | 11% | -0.0 |
| **COPD** |  | 59 (7.0%) | 30 (8.0%) | 29 (6.2%) | -0.070 |  | 11% | 11% | 0.0 |
| **Vasopressor** |  |  |  |  |  |  |  |  |  |
| Noradrenaline |  | 734 (86.7%) | 287 (76.1%) | 447 (95.1%) | 0.561 |  | 87% | 89% | 0.060 |
| Dopamine |  | 169 (20.0%) | 107 (28.4%) | 62 (13.2%) | -0.381 |  | 17% | 15% | -0.079 |
| Dobutamine |  | 132 (15.6%) | 54 (14.3%) | 78 (16.6%) | 0.063 |  | 11% | 17% | 0.17 |
| **Supplemental oxygen** |  | 256 (30.2%) | 105 (27.9%) | 151 (32.1%) | 0.093 |  | 35% | 29% | -0.124 |
| **NHF** |  | 51 (6.0%) | 27 (7.2%) | 24 (5.1%) | -0.086 |  | 10% | 9% | -0.033 |
| **NPPV** |  | 19 (2.2%) | 15 (4.0%) | 4 (0.9%) | -0.205 |  | 5% | 2% | -0.16 |
| **Mechanical Ventilation** |  | 628 (74.1%) | 290 (76.9%) | 338 (71.9%) | -0.115 |  | 66% | 78% | 0.26 |
| **Antibiotics** |  |  |  |  |  |  |  |  |  |
| multiple antibiotics |  | 548 (64.7%) | 226 (59.9%) | 322 (68.5%) | 0.179 |  | 58% | 63% | 0.099 |
| Ampicillin/sulbactam |  | 145 (17.1%) | 56 (14.9%) | 89 (18.9%) | 0.109 |  | 21% | 16% | -0.15 |
| Tazobactam/piperacillin or Tazobactam /ceftolozane |  | 285 (33.6%) | 128 (34.0%) | 157 (33.4%) | -0.012 |  | 35% | 37% | 0.040 |
| Third-generation cephalosporin, poor activity against Pseudomonas |  | 243 (28.7%) | 99 (26.3%) | 144 (30.6%) | 0.097 |  | 23% | 28% | 0.111 |
| Carbapenem |  | 351 (41.4%) | 150 (39.8%) | 201 (42.8%) | 0.060 |  | 38% | 43% | 0.099 |
| Fluoroquinolone |  | 165 (19.5%) | 76 (20.2%) | 89 (18.9%) | -0.031 |  | 19% | 18% | -0.025 |
| Macrolide |  | 226 (26.7%) | 95 (25.2%) | 131 (27.9%) | 0.061 |  | 26% | 29% | 0.065 |
| Anti-MRSA drug |  | 128 (15.1%) | 37 (9.8%) | 91 (19.4%) | 0.273 |  | 7% | 12% | 0.168 |
| **Hydrocortisone equivalent corticosteroid volume** |  | 920.9 (1529.4) | 1767.9 (1988.5) | 241.6 (101.6) | -1.084 |  | 283 | 307 | 0.188 |
| **ICU** |  | 423 (49.9%) | 146 (38.7%) | 277 (58.9%) | 0.412 |  | 43% | 50% | 0.136 |
| **HDU** |  | 285 (33.6%) | 135 (35.8%) | 150 (31.9%) | -0.082 |  | 39% | 34% | -0.101 |

mPSL; methylprednisolone, JCS; Japan coma scale, COPD; chronic obstructive pulmonary disease, NHF; nasal high flow therapy, NPPV; noninvasive positive pressure ventilation, ICU; intensive care unit, HDU; high dependency unit.

Supplementary Table 3. Baseline characteristics and comorbidities of the subgroup of patients without shock

|  |  | Before matching | | | |  | After matching | | |
| --- | --- | --- | --- | --- | --- | --- | --- | --- | --- |
|  |  | Total | mPSL | Hydrocortisone | Standardised difference |  | mPSL | Hydrocortisone | Standardised difference |
|  |  | N=4,237 | N=3,625 | N=612 |  |  | N= 493 | N=493 |  |
| **Age** |  | 81.6 (8.0) | 81.5 (8.0) | 82.1 (8.3) | 0.070 |  | 83.1 | 82.1 | 0.008 |
| **male** |  | 3,119 (73.6%) | 2,715 (74.9%) | 404 (66.0%) | -0.196 |  | 68% | 69% | 0.009 |
| **Fiscal year** |  |  |  |  |  |  |  |  |  |
|  | 2017 | 1,117 (26.4%) | 937 (25.8%) | 180 (29.4%) | 0.080 |  | 30% | 27% | -0.059 |
|  | 2018 | 1,113 (26.3%) | 949 (26.2%) | 164 (26.8%) | 0.014 |  | 28% | 27% | -0.018 |
|  | 2019 | 964 (22.8%) | 818 (22.6%) | 146 (23.9%) | 0.031 |  | 21% | 26% | 0.124 |
|  | 2020 | 539 (12.7%) | 473 (13.0%) | 66 (10.8%) | -0.070 |  | 11% | 11% | 0.007 |
|  | 2021 | 504 (11.9%) | 448 (12.4%) | 56 (9.2%) | -0.104 |  | 11% | 9% | -0.069 |
| **Hospital Volume** |  | 140.2 (108.7) | 142.3 (111.7) | 128.1 (87.7) | -0.141 |  | 127 | 129 | 0.025 |
| **Smoking** | Non-Smoker | 1,793 (42.3%) | 1,453 (40.1%) | 340 (55.6%) | 0.313 |  | 49% | 51% | 0.045 |
|  | Smoker | 1,879 (44.3%) | 1,683 (46.4%) | 196 (32.0%) | -0.298 |  | 41% | 36% | -0.113 |
|  | Missing | 565 (13.3%) | 489 (13.5%) | 76 (12.4%) | -0.032 |  | 10% | 13% | 0.102 |
| **JCS** | 0 | 2,884 (68.1%) | 2,512 (69.3%) | 372 (60.8%) | -0.179 |  | 65% | 64% | -0.030 |
|  | 1-3 | 1,041 (24.6%) | 875 (24.1%) | 166 (27.1%) | 0.068 |  | 24% | 25% | 0.028 |
|  | 10-30 | 197 (4.6%) | 149 (4.1%) | 48 (7.8%) | 0.158 |  | 6% | 8% | 0.055 |
|  | 100-300 | 115 (2.7%) | 89 (2.5%) | 26 (4.2%) | 0.10 |  | 4% | 3% | -0.065 |
| **A-DROP score** | 3 | 3,248 (76.7%) | 2,813 (77.6%) | 435 (71.1%) | -0.15 |  | 70% | 76% | 0.128 |
|  | 4 | 874 (20.6%) | 724 (20.0%) | 150 (24.5%) | 0.109 |  | 24% | 21% | -0.082 |
|  | 5 | 115 (2.7%) | 88 (2.4%) | 27 (4.4%) | 0.109 |  | 5% | 3% | -0.109 |
| **Asthma** |  | 964 (22.8%) | 847 (23.4%) | 117 (19.1%) | -0.104 |  | 19% | 22% | 0.071 |
| **COPD** |  | 589 (13.9%) | 541 (14.9%) | 48 (7.8%) | -0.224 |  | 10% | 10% | -0.027 |
| **Supplemental oxygen** |  | 3,296 (77.8%) | 2,821 (77.8%) | 475 (77.6%) | -0.005 |  | 77% | 78% | 0.044 |
| **NHF** |  | 337 (8.0%) | 302 (8.3%) | 35 (5.7%) | -0.102 |  | 6% | 6% | -0.009 |
| **NPPV** |  | 78 (1.8%) | 68 (1.9%) | 10 (1.6%) | -0.018 |  | 2% | 1% | -0.064 |
| **Mechanical Ventilation** | | 661 (15.6%) | 596 (16.4%) | 65 (10.6%) | -0.171 |  | 0.14 | 14% | 12% |
| **Antibiotics** |  |  |  |  |  |  |  |  |  |
| multiple antibiotics |  | 1,666 (39.3%) | 1,443 (39.8%) | 223 (36.4%) | -0.069 |  | 33% | 36% | 0.051 |
| Ampicillin/sulbactam |  | 1,182 (27.9%) | 982 (27.1%) | 200 (32.7%) | 0.122 |  | 34% | 32% | -0.039 |
| Tazobactam/piperacillin or Tazobactam /ceftolozane |  | 965 (22.8%) | 823 (22.7%) | 142 (23.2%) | 0.012 |  | 20% | 22% | 0.045 |
| Third-generation cephalosporin, poor activity against Pseudomonas |  | 1,429 (33.7%) | 1,249 (34.5%) | 180 (29.4%) | -0.108 |  | 30% | 32% | 0.057 |
| Carbapenem |  | 635 (15.0%) | 543 (15.0%) | 92 (15.0%) | 0.001 |  | 15% | 13% | -0.041 |
| Fluoroquinolone |  | 577 (13.6%) | 509 (14.0%) | 68 (11.1%) | -0.088 |  | 10% | 11% | 0.026 |
| Macrolide |  | 755 (17.8%) | 667 (18.4%) | 88 (14.4%) | -0.109 |  | 13% | 16% | 0.081 |
| Anti-MRSA drug |  | 49 (1.2%) | 32 (0.9%) | 17 (2.8%) | 0.142 |  | 2% | 2% | 0.0 |
| **Hydrocortisone equivalent corticosteroid volume** |  | 1071.5 (1493.4) | 1213.7 (1569.3) | 229.3 (153.9) | -0.883 |  | 268 | 248 | -0.131 |
| **ICU** |  | 253 (6.0%) | 213 (5.9%) | 40 (6.5%) | 0.027 |  | 7% | 5% | -0.069 |
| **HDU** |  | 846 (20.0%) | 740 (20.4%) | 106 (17.3%) | -0.079 |  | 19% | 18% | -0.031 |

mPSL; methylprednisolone, JCS; Japan coma scale, COPD; chronic obstructive pulmonary disease, NHF; nasal high flow therapy, NPPV; noninvasive positive pressure ventilation, ICU; intensive care unit, HDU; high dependency unit.
